# Death of Dr. Forry

**Published:** 1844-12

**Authors:** 


### DEATH OF DR. SAMUEL FORRY.

*Died*—In New York, on Saturday, the 9th ult., in the 33d year of his age, Dr. SAMUEL FORRY, Editor of the New York Journal of Medicine.

We must mingle our regret and sorrow with those of Dr. Forry's immediate friends; his death is a loss to the whole profession. Although we never looked upon him, although we did not even know him, save as an author and an editor, still he seemed bound to us—we scarce know why—by more than ordinary ties. His unwearied industry, his earnest, truthful, and whole-hearted devotion to the cause of science, had won him a home in the affections of all his brethren. Young as he was, Dr. Forry had already attained to eminence. His work on the climate of the United States made his name widely known both at home and abroad, and will long remain as a monument of his talents and industry. His career as editor, though short, was full of promise, and had he lived, his journal would have been among the first in the country. These, however, are but a small portion of his labors; his mind and pen were never idle; he was eager in the pursuit of facts, and his ready pen was constant in its work. Besides his labors in the more direct line of the profession, Dr. Forry was a welcome contributor to some of the leading American periodicals. The North American Review and the Democratic Review, are both indebted to him, if we mistake not, for some valuable papers. "But that noble tree will never more bear fruit or blossom!" C.

